# Improvement of diagnosis in children with Burkitt lymphoma in Kenya: feasibility study for the implementation of fluorescence *in situ* hybridisation testing for *MYC* and the *MYC/IGH* translocation

**DOI:** 10.3332/ecancer.2023.1505

**Published:** 2023-02-08

**Authors:** Gail H Vance, Teresa Lotodo, Nicholas Kigen, Ryan Stohler, Haki Choi, Festus Njuguna, Ann M Moormann, Erastus Kirwa, Sandra Langat, Patrick Loehrer, Terry Vik

**Affiliations:** 1 Department of Medical and Molecular Genetics, Indiana University School of Medicine, Indianapolis, IN 46202, USA; 2 Department of Pathology, Moi University, Eldoret, Kenya; 3 AMPATH Reference Laboratory, Eldoret, Kenya; 4 Department of Child Health and Pediatrics, Moi University, Eldoret, Kenya; 5 Department of Medicine, University of Massachusetts Chan Medical School, Worcester, MA 01655, USA; 6 Kenya Medical Research Institute (KEMRI), Kisumu, Kenya; 7 AMPATH Pediatric Oncology, Eldoret, Kenya; 8 Department of Medicine, Indiana University School of Medicine, Indianapolis, IN 46202, USA; 9 Department of Pediatrics, Indiana University School of Medicine, Indianapolis, IN 46202, USA

**Keywords:** Burkitt lymphoma, fluorescence in situ hybridisation, FISH, MYC/IGH translocation, MYC rearrangement

## Abstract

**Background:**

Indiana University (IU) initiated fluorescence in situ hybridisation (FISH) methodology for Burkitt Lymphoma (BL) to advance the accuracy and speed of diagnosis in the AMPATH Reference Laboratory at Moi Teaching and Referral Hospital (MTRH) in Eldoret, Kenya. Standard diagnostic testing for BL at MTRH includes morphology of the biopsy specimen or aspirate and limited immunohistochemistry panels.

**Methods:**

Tumour specimens from 19 children enrolled from 2016 to 2018 in a prospective study to improve the diagnosis and staging of children with suspected BL were evaluated. Touch preps from biopsy specimens or smears from fine needle aspiration were collected, stained with Giemsa and/or H&E and reviewed by pathologists to render a provisional diagnosis. Unstained slides were stored and later processed for FISH. Duplicate slides were split between two laboratories for analysis. Flow cytometry results were available for all specimens. Results from the newly established FISH laboratory in Eldoret, Kenya were cross-validated in Indianapolis, Indiana.

**Results:**

Concordance studies found 18 of 19 (95%) of specimens studied yielded analysable FISH results for one or both probe sets (*MYC* and *MYC/IGH*) in both locations. There was 94% (17/18) concordance of results between the two FISH laboratories. FISH results were 100% concordant for the 16 specimens with a histopathological diagnosis of BL and two of three non-BL cases (one case no result in IU FISH lab). FISH was similarly concordant with flow cytometry for specimens with positive flow results with the exception of a nasopharyngeal tumour with positive flow results for CD10 and CD20 but was negative by FISH. The modal turn-around time for FISH testing on retrospective study specimens performed in Kenya ranged between 24 and 72 hours.

**Conclusion:**

FISH testing was established, and a pilot study performed, to assess the feasibility of FISH as a diagnostic tool for the determination of BL in a Kenyan paediatric population. This study supports FISH in limited resource settings to improve the accuracy and speed of diagnosis of BL in Africa.

## Introduction

Burkitt lymphoma (BL) is a common paediatric cancer representing greater than 50% of childhood cancers in sub-Saharan Africa. A subtype of non-Hodgkin lymphoma (non-HL), endemic BL (eBL) is associated with Epstein–Barr virus and continued exposure to *Plasmodium falciparum* malaria infections [1, 2]. A highly aggressive tumour, delay of diagnosis results in delay of treatment, resulting in high morbidity and mortality [3–5].

Fluorescence *in situ* hybridisation (FISH) is a molecular cytogenetic tool for identifying recurring translocations in haematological malignancies [6, 7]. In BL, the t(8;14)(q24;q32) is the characteristic cytogenetic translocation involving the juxtaposition of *MYC* with *IGH* on chromosome 14 resulting in *MYC* dysregulation [8, 9]. Probes flanking the *MYC* and *IGH* breakpoints create a dual (or tri) colour, dual fusion signal pattern in BL cells containing the translocation. Disruption of the *MYC* gene locus may also be identified with a break apart probe strategy.

This FISH diagnostic pilot study was initiated in the AMPATH Reference Laboratory (ARL) at Moi Teaching and Referral Hospital (MTRH) in Eldoret, Kenya and supported by an Administrative Supplement (PA-16-086) to an active clinical trial with the aim to complement and improve histological diagnosis of a core needle biopsy or fine needle aspiration (FNA) of the tumour. Eligibility for the clinical trial included a clinical suspicion of BL in a child 0–13 years of age. Children with prior treatment of cancer were excluded. The trial was approved by Institutional Review Boards of Indiana University (IU) and MTRH. Parental consent was obtained prior to study entry.

## Materials and methods

From 2016 through 2018, 96 children of 0–13 years with clinical suspicion of BL were recruited at MTRH in Eldoret, Kenya. A tissue biopsy or FNA, or both, was obtained from the child’s tumour. Tissue was collected from various MTRH locations including the surgical suite, radiology, dental services and others. The biopsy tissue was generally divided into two pieces with one piece placed in formalin for histological diagnosis and another biopsy core was touched to positively charged slide(s) and taken to the ARL. FNA aspirates were prepared in the procedure room. Aspirate material was smeared on slides for histological review and also deposited into Roswell Park Memorial Institute (RPMI) media for flow cytometry. FNA smears were stained with Giemsa and/or H&E and reviewed by pathologists to render a histopathological diagnosis. Flow cytometry was performed on a BD FACS Calibur in ARL. The sample was subjected to a leukaemia/lymphoma panel that included CD10, CD19, CD20, kappa and lambda. For the purposes of this study, a value of ≥20% for CD10, CD19 or CD20 was considered significant. Additional unstained slides, when available, were stored for future studies.

**Figure 1.**
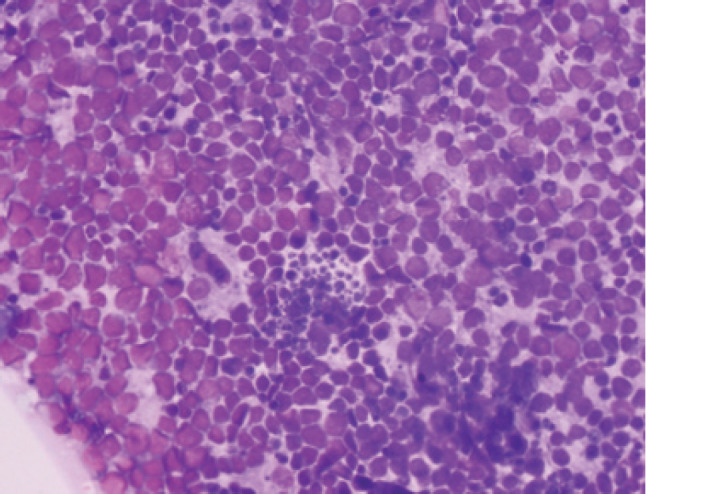
Tumour from MBL-019: abdominal mass, core biopsies. Sections show a tumour composed of sheets of intermediate cells with scant cytoplasm, irregular hyperchromatic nuclei, inconspicuous nucleoli and brisk mitotic activity. Focal area exhibits a ‘starry sky’ appearance. Morphologic features consistent with BL.

A total of 96 children were enrolled and 43 cases of BL were confirmed by histopathology on biopsy specimens (Figure 1). Of materials collected, initially for flow, then FISH, FISH required two slides for testing in the two FISH laboratories. Concordance studies were performed between the ARL and IU Cytogenetics FISH laboratories. Stored slides representing 19 patient tumours were interrogated by FISH to assess feasibility and accuracy of testing. Cases with duplicate unstained slides from either a tissue biopsy touch prep or FNA were used for FISH and split between the two institutions. Slides had been stored in slide boxes, exposed to ambient air temperature prior to FISH testing. Once the study slides were selected, they were immersed in 3:1 methanol:acetic acid fixative for 20 minutes and air-dried. Slides processed in the IU FISH laboratory were transported from Eldoret in plastic slide carriers and placed in checked luggage until arrival in the FISH laboratory and immediately immersed in fixative.

**Figure 2.**
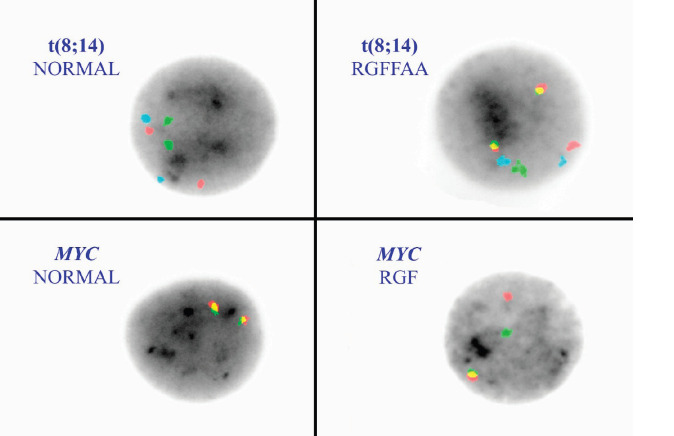
FISH on interphase nuclei with the t(8;14) and *MYC* break apart probes. The left upper corner demonstrates a normal FISH pattern (left) for the t(8:14) with RRGGAA. Red = *MYC*, Green = *IGH* and aqua (A) is the chromosome 8 centromere. The right upper corner is the abnormal dual fusion signal pattern representing a t(8;14). The lower panels show a normal *MYC* break apart probe pattern (fusion/fusion signal pattern) on the left and a rearrangement pattern RGF on the right.

The most common cytogenetic translocation associated with BL and found in approximately 85% of the BL is the t(8;14) involving the *MYC* gene on chromosome 8 and the *IGH* gene on chromosome 14. Probes for *MYC* and *IGH* were utilised. The tri-colour, dual fusion probe set (Figure 2) included probes that hybridise to *MYC* (red), *IGH* (green) and the chromosome 8 centromere (aqua) (LSI IGH/MYC/CEP 8 Tri-color Dual Fusion Probe Kit-Abbott Molecular, Des Plaines, IL). There are also two variant BL translocations, t(2;8)(p12;q24) and t(8;22)(q24;q11.2) involving *IGK* (15%) and *IGL* (5%) of BL cases, respectively. Therefore, we also incorporated a *MYC* break apart strategy (LSI MYC Dual Color Break Apart Rearrangement Probe-Abbott Molecular) to confirm the t(8;14) and potentially detect variant translocations (Figure 2) in the cases with negative t(8;14) results but positive by flow cytometry and with a histopathological diagnosis of BL. The *MYC* probe is disrupted with a rearrangement of *MYC* to any partner locus; 5’ segment of *MYC* (red) and 3’ segment (green) split to yield a red/green/fusion (RGF) rearrangement pattern.

Interphase FISH was performed following a standard, shared protocol using direct-labelled probes. Briefly, unstained touch prep and FNA slides were fixed in 3:1 methanol:acetic acid and allowed to air dry. They were pre-treated in 2× saline sodium citrate buffer (SSC) followed by 15 minutes of 0.005% pepsin, both at 37°C. Denaturation, ethanol dehydration and hybridisation with the LSI MYC and LSI IGH/MYC, CEP 8 probes were carried out as per manufacturer’s instructions. Cells were counterstained with DAPI (4’,6,diamidino-2-phenylindole) and observed under a Leica DM2000 LED fluorescence microscope. One hundred cells scored for each probe set at the respective laboratory.

A haematopathologist (TL) and one lab scientist (NK) spent 2 weeks in the IU Cytogenetics FISH laboratory training on FISH protocols, learning FISH microscopy and FISH result interpretation. The IU Cytogenetics FISH laboratory is Clinical Laboratory Improvement Amendments (CLIA)-certified and accredited by the College of American Pathologists. The Leica fluorescence microscope with appropriate filters and imaging capabilities was purchased in the U.S. and established in laboratory space donated by the ARL in 2017, midway during the clinical trial. FISH probes were brought from the US and other reagents purchased via the AMPATH research office. A study investigator (GHV) travelled to Eldoret to oversee initial lab setup and develop the IU Cytogenetics FISH testing protocols in the ARL.

## Results

Nineteen specimens were studied in the AMPATH and IU FISH laboratories. Results for one or two probe sets were obtained in both laboratories for 18/19 specimens (95%) (Table 1). Concordant results occurred in 17 of 18 cases (94%) with a disagreement of the t(8;14) result for study specimen MBL12. A t(8;14) signal pattern was identified in the ARL but not in the IU FISH laboratory although the IU lab did detect a *MYC* rearrangement. There were no results for *MYC* from the ARL for this specimen and additional slides to repeat the testing were not available.

Sixteen of the 19 specimens had a histological diagnosis of BL (Table 2). The three non-BL tumours included MBL-33, MBL-63 and MBL-95 classified as nodular variant of HL (MBL-33) and nasopharyngeal cancer (MBL-63, MBL-95). FISH results from both laboratories were positive/concordant for the t(8;14) or *MYC* rearrangement in all 16 histological cases of BL cases and negative for either the t(8;14) and/or MYC probe in the three non-BL cases although the IU lab had indeterminate results for both probes for specimen MBL-095 and no signal (NS) for the t(8;14) for MBL-63.

The 16 BL specimens also had positive flow results indicating concordance with histological diagnosis, flow cytometry and FISH for these specimens. Flow cytometry results for CD19 and CD20 were also positive for MBL-33, the nodular lymphocyte-predominant variant of HL (NLPHL).

Specimen MBL-061 was positive for *MYC* rearrangement only in both labs indicating it may represent a variant BL t(2;8) or t(8;22) or another aggressive lymphoma with *MYC* rearrangement. Five specimens (MBL-012, 048, 050, 057, 058) studied in the AMPATH FISH laboratory were unsuccessful for *MYC* detection although a *MYC* rearrangement was detected on the same specimens in the IU FISH laboratory. It was determined that the placement of the *MYC* probe was too far to the right of the optimal cellular area for successful microscopic FISH analysis. Subsequent adjustment was made to load both probe sets closer to the frosted edge of the slide and to mark the slide for probe placement based on a microscopic pre-screen of the slide. Additional slides for these tumours were not available for re-analysis of *MYC*.

## Discussion

FISH is a powerful diagnostic tool for screening, early detection, tumour classification and monitoring the efficacy of interventions in leukaemia and lymphoma [8, 10]. Metaphase preparations from cultured cells are often considered the ‘gold standard’ because chromosome morphology and position of the fluorescent signals may be visualised directly. However, a major advantage of FISH is that it can also be performed on non-dividing interphase cells. Interphase nucleus assessment from uncultured preparations allows for a rapid screening for specific chromosome rearrangements or numerical abnormalities associated with haematologic malignancies [7].

In this pilot study, a FISH laboratory was established in Kenya and interphase FISH testing was introduced on uncultured biopsy touch prep specimens and FNA smears from suspected BL patients enrolled in a clinical trial. Concordance studies were performed between the ARL and IU Cytogenetics FISH laboratories utilising probe sets for the t(8;14) and *MYC*.

Eighteen of the 19 (95%) specimens studied yielded analysable FISH results for one or both probe sets in both locations. The IU FISH lab was unable to obtain results for either probe set on MBL-095. There was 94% (17/18) concordance of results between the two FISH laboratories demonstrating the feasibility of performing FISH for BL in the ARL. Further, we demonstrate concordance of FISH with a histological diagnosis of BL and positive flow cytometry results. One non-Burkitt specimen (MBL-33) had positive flow results for CD19 and CD20. This specimen was determined to be a NLPHL. NLPHL is a morphologically related subtype, but clinically and pathogenetically distinct from classic HL with large and atypical lymphocyte-predominant cells that express B-cell markers such as CD20+ [11, 12].

To improve the accuracy of FISH in diagnosis of BL, two probe strategies were utilised, a tri-colour, dual fusion strategy for the t(8;14) and a break apart strategy for *MYC*. For the 16 specimens with a histological diagnosis of BL, positive results with the t(8;14) probe set were obtained for 13/16 (81%) in the ARL lab compared to 14/16 (87.5%) in the IU FISH lab. Both labs showed negative results for the translocation for MBL-61 with Burkitt histology and positive *MYC* FISH results. This specimen was thought to represent a variant BL translocation. There were no results for the t(8;14) in specimens MBL-034 and MBL-072 in the ARL lab and the t(8;14) was negative for MBL-012 in the IU FISH lab, yielding discordant results with the ARL lab FISH, histological diagnosis and flow results. The IU FISH lab had 100% concordance with histological diagnosis and flow results for the *MYC* probe and the 16 BL specimens compared to 69% for the ARL lab primarily due to laboratory error in probe placement. The IU FISH laboratory had no results for either the t(8;14) or *MYC* probe for specimen MBL-95. The quality of this specimen may have been affected by prolonged storage and shipment to Indianapolis.

Noting that positive *MYC* FISH results alone are not sufficient to support a diagnosis of BL, Troxell *et al* [13] reported on seven clinically or morphologically suspicious cases for BL. Six of seven were positive for *MYC* rearrangement by FISH but only 3/6 represented BL with one designated as an atypical BL. The study illustrated the value of FNA in diagnosis of BL as well as the importance of integrating results of ancillary studies noting that *MYC* may be rearranged in other aggressive lymphomas. A similar study by Chen *et al* [14], looking at clinical pathological analysis of B-cell lymphomas concluded that the use of two probe sets with *MYC* rearrangement and *MYC/IGH* fusion was detected in 94.2% (81/86) and 83.7% (72/86) cases of BL. Our study, like that of Chen *et al* [14], utilised the *MYC* rearrangement probe and the probe set for the typical BL translocation. We also included flow cytometry with a panel of antibodies for recognition of B-cell neoplasia thus supporting the sensitivity and accuracy of our FISH results for BL.

FISH testing was also undertaken to improve the speed of BL diagnosis. Njuguna *et al* [15] studied the time lag and other factors influencing the time to diagnosis and start of treatment among paediatric oncology patients in Kenya. For that study, participants were enrolled at MTRH from August 2013 to July 2014. Diagnostic delay was defined as the time from the onset of symptoms to diagnosis. The time of diagnosis from arrival at MTRH was reported to be approximately 11 days. For this current study conducted from November 2016 to March 2018, specimens were retrieved from several MTRH locations and prepared for flow cytometry and FISH. Once received in the ARL FISH laboratory, FISH results with the *MYC* and *MYC/IGH* probe sets were available within 24–72 hours, similar to FISH testing turnaround times in other international laboratories.

A limitation of our study was the identification of two or more unstained slides available for FISH in both laboratories reducing the total number of study specimens. Further, the slides were stored in ambient temperature and not fixed with a methanol/acetic acid solution after collection allowing for degradation of the cells. A variant BL translocation including a t(2;8) or t(8;22) was suspected for MBL-061; however, further definitive testing for these translocations was not included in our study protocol.

## Conclusion

Here we demonstrate the development of FISH as a diagnostic tool for BL in Eldoret, Kenya. To the authors’ knowledge, FISH testing in the ARL is the only FISH testing performed for cancer in Kenya. The study achieved the objectives of establishing a FISH laboratory and training personnel for successful performance of FISH testing, thus reducing the diagnostic interval from tissue procurement to BL diagnosis. Introducing FISH for a more conclusive diagnosis of BL and other haematological disorders in an under-resourced public health facility is a major milestone. It shows that major strides are being made to include ancillary tests like immunophenotyping, cytogenetics and molecular tests in diagnosis of haematological disorders as recommended by the World Health Organization.

Our study to assess the utility of FISH to improve time to diagnosis of eBL in this population was a feasibility study to test whether it was even possible to use this test in a low and middle income country (LMIC). FISH testing performed in this study was supported by grants and academic funds. Going forward, it would be important for the Kenyan National Health Insurance Fund to consider coverage for testing. We plan to introduce FISH into standard clinical study protocols and to assess potential changes in the clinical outcomes for the study participants including reducing time from symptom onset to diagnosis and initiation of therapy.

## Authors’ contributions

Conceptualisation and study design: GHV, TL, FN, AMM, PL, TV

Technical work and data collection: GHV, TL, NK, RS, HC, EK, TV

Data management: GHV, TL, NK, RS, HC, SL

Writing: GHV, TL, AMM, TV

All authors read and approved the manuscript in its current form.

## Conflicts of interest

The authors declare that they have no competing interests.

## Figures and Tables

**Table 1.** Summary of FISH study results.

Overall concordance with FISH	AMPATH t(8;14)	IU t(8;14)	AMPATH MYC	IU MYC	Not concordant
Positive cases	13/19	14/19	11/ 19	16/19	MBL-012 (8;14)
Negative cases	4/19	3/19	3/19	2/19	
No signal (NS)	2 NS	2 NS	5 NS	1 NS	

**Table 2.** Study subjects: clinical information and laboratory results.

Study ID/specimen type	Age	Gender	Tumour site	Histology	Flow (CD19, CD20, CD10)	FISH (AMPATH) t(8;14)/*MYC*	FISH (IU) t(8;14)/*MYC*
MBL.008 Touch print	5 years	Male	Abdomen	BL	CD19-82% (+) CD20-72% (+) CD10-82% (+)	+/+	+/+
MBL .012^a^ FNA	6 years	Male	Right buccal	BL	CD19-70% (+) CD20-68% (+) CD10-70% (+)	+/NS	−/+
MBL.013 FNA	9 years	Female	Jaw	BL	CD19-87% (+) CD20-87% (+) CD10-82% (+)	+/+	+/+
MBL.018 Touch print	15 years	Male	Abdominal	BL	CD19-83% (+) CD20-83% (+) CD10-83% (+)	+/+	+/+
MBL.019 Touch print	4 years	Male	Abdominal	BL	CD19-58% (+) CD20-58% (+) CD10-58% (+)	+/+	+/+
MBL.025 Touch print	3 years	Female	Abdominal	BL	CD19-32% (+) CD20-44% (+) CD10-32% (+)	+/+	+/+
MBL.033 Touch print	12 years	Male	Neck	HL (nodular sclerosis variant)	CD19-25% CD20-25% CD10-<1%	−/−	−/−
MBL.034 Touch print	6 years	Male	Jaw	BL	CD19-88% (+) CD20-88% (+) CD10-84% (+)	NS/+	+/+
MBL.044 Touch print	3 years	Male	Abdominal	BL	CD19-71% (+) CD20-57% (+) CD10-75% (+)	+/+	+/+
MBL.048 Touch print	5 years	Male	Abdominal	BL	CD19-42% (+) CD20-50% (+) CD10-56% (+)	+/NS	+/+
MBL.050 Touch print	6 years	Male	Jaw	BL	CD19-58% (+) CD20-62% (+) CD10-60% (+)	+/NS	+/+
MBL.057 Touch print	8 years	Female	Abdominal	BL	CD19-47% (+) CD20-47% (+) CD10-48% (+)	+/NS	+/+
MBL.058 Touch print	6 years	Female	Abdominal	BL	CD19-73% (+) CD20-72% (+) CD10-72% (+)	+/NS	+/+
MBL.061 FNA	11 years	Male	Abdominal	BL	CD19-45% (+) CD20-47% (+) CD10-47% (+)	−/+	−/+
MBL.063 Touch print	14 years	Male	Neck	Naso-pharyngeal cancer	CD19-10% CD20-7% CD10-7%	−/−	NS/−
MBL.064 Touch print	5 years	Male	Maxilla	BL	CD19-73% (+) CD20-72% (+) CD10-72% (+)	+/+	+/+
MBL.072 Touch print	7 years	Female	Jaw	BL	CD19-71% (+) CD20-51% (+) CD10-71% (+)	NS/+	+/+
MBL.082 Touch print	13 years	Male	Jaw	BL	CD19-70% (+) CD20-70% (+) CD10-57% (+)	+/+	+/+
MBL.095 Touch print	8 years	Male	Jaw	Naso-pharyngeal cancer	CD19-13% (+) CD20-1% (+) CD10-2% (+)	−/−	NS

a Discordant result between ARL and IU FISH laboratories
